# Reconstructing the female reproductive system using 3D bioprinting in tissue engineering

**DOI:** 10.1016/j.mtbio.2025.102127

**Published:** 2025-07-22

**Authors:** Heesuh Yi, Gaeun Lee, Sanghyeok Park, Juhyeong Ha, Dayeong Choi, Jihoon Ko, Jungho Ahn

**Affiliations:** a Department of Chemical and Biological Engineering, Gachon University, Seongnam-si, Gyeonggi-do, 13120, Republic of Korea; b Department of MetaBioHealth, Sungkyunkwan University, Suwon, Gyeonggi-do, 16419, Republic of Korea; c Department of Biophysics, Institute of Quantum Biophysics, Sungkyunkwan University, Suwon, Gyeonggi-do, 16419, Republic of Korea; d Department of BioNano Technology, Gachon University, Seongnam-si, Gyeonggi-do, 13120, Republic of Korea

## Abstract

Three-dimensional bioprinting enables the precise fabrication of complex biological tissues through the layer-by-layer deposition of living cells and biomaterials, offering a promising strategy for reconstructing the female reproductive system. This technology has facilitated the development of in vitro models for tissues such as the endometrium, ovary, cervix, and vagina, providing improved structural fidelity and functional relevance. By leveraging bioinks, including decellularized extracellular matrix and advanced bioprinting techniques, researchers can recreate the intricate microarchitectures and vascular networks required for tissue functionality. These bioprinted systems serve as high-fidelity microphysiological systems for studying reproductive health, modeling disease progression, and evaluating therapeutic responses. Moreover, the integration of artificial intelligence into bioprinting workflows enhances reproducibility, scalability, and patient-specific customization. This review summarizes recent advances in reproductive tissue bioprinting and highlights its potential to transform regenerative gynecology and personalized reproductive healthcare.

## Graphical abstract

**Figure:**
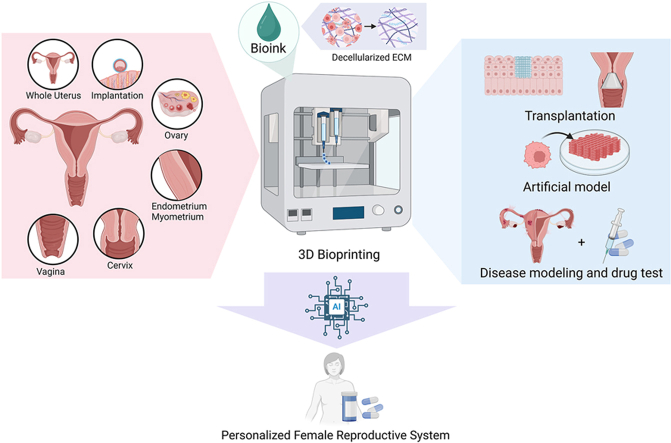

## 1 Introduction

The female reproductive system is a complex network of organs and tissues—including the ovaries, fallopian tubes, uterus, cervix, and vagina—that work together to ensure fertility, hormonal regulation, and reproductive health [1]. The ovaries produce both oocytes and key sex hormones, such as estrogen and progesterone (P4), which regulate the menstrual cycle and induce cyclical remodeling of the endometrium. After ovulation, the oocyte is typically fertilized in the fallopian tube and transported to the uterus, where the hormonally primed endometrium facilitates embryo implantation. If implantation fails, hormonal withdrawal triggers the shedding of the endometrial lining during menstruation [2]. The uterus, particularly its inner lining called the endometrium, undergoes proliferative and secretory phases under the influence of ovarian hormones, creating an optimal environment for embryo implantation and fetal development; if implantation does not occur, hormone withdrawal leads to the shedding of the functional endometrial layer during menstruation [3]. The cervix connects the uterus to the vagina, regulates the passage of sperm and menstrual blood, and produces mucus whose properties change with hormonal fluctuations, while also forming a barrier against pathogens during pregnancy [4]. The vagina is a fibromuscular canal that serves as the site for sperm deposition, the birth canal during delivery, and a protective barrier against external microorganisms, with its epithelium maintained by estrogen [5]. These organs are highly dynamic, with their structure and function tightly regulated by cyclical endocrine signals, cellular interactions, and extracellular matrix (ECM) remodeling, all of which are essential for reproductive success. Understanding these complex biological processes is critical for the rational design of engineered tissue models that aim to recapitulate native female reproductive function [6,7].

Reproduction is a vital biological process essential for species survival, involving the coordinated interaction of specialized tissues, organs, and hormones. In the female reproductive system, hormones such as estradiol (E2) and P4 regulate the proliferative and secretory phases of the endometrium, creating an optimal environment for implantation and fetal development. The luteinizing hormone (LH) surge triggers ovulation, enabling fertilization in the fallopian tubes and subsequent zygote transport to the uterus. During pregnancy, the cervix serves as a protective barrier, safeguarding against external microorganisms. Recent advancements in reproductive biology have deepened our understanding of these processes, offering critical insights into tissue development and function [6,7].

The study of human reproduction requires a multidisciplinary approach. While animal models and two-dimensional (2D) cell cultures have advanced reproductive biology research, they present significant limitations. Animal models often fail to replicate human reproductive physiology accurately [8,9], and 2D models cannot capture the dynamic, three-dimensional (3D) complexity of living tissues. These challenges not only highlight the urgent need for advanced in vitro platforms but also underscore the critical importance of evaluating how far current strategies have come and where they continue to fall short.

3D bioprinting, an emerging technology that precisely combines living cells with biomaterials, offers a solution for addressing these limitations. By recreating the intricate architecture and functionality of native tissues, bioprinting surpasses conventional tissue-engineering approaches [10]. This technology has rapidly evolved, incorporating methods such as extrusion-based, inkjet-based, laser-assisted and Vat-polymerization-based bioprinting to improve precision and cell viability [11]. Bioprinting's ability to spatially organize multiple cell types and biomaterials enables the creation of tissue models with high structural and functional fidelity [12], providing valuable tools for regenerative medicine, drug testing, and disease modeling [[13], [14], [15]].

Successful bioprinting of female reproductive tissues requires careful consideration of several design criteria: (1) Hormonal responsiveness, to mimic physiological responses to estrogen and P4; (2) Matrix composition, reflecting the tissue-specific ECM for cell support and signaling; (3) Vascularization, ensuring nutrient and oxygen supply in thick constructs; (4) Multi-cellularity, to recapitulate the complex cellular architecture; and (5) Mechanical properties, matching the elasticity and strength of native tissues. These criteria are critical for developing physiologically relevant and functional bioprinted constructs.

In reproductive medicine, 3D bioprinting has demonstrated significant potential in addressing critical challenges, including restoring ovarian function, regenerating the endometrium, and reconstructing vaginal tissue. Regulatory advancements, such as the FDA Modernization Act 2.0/3.0, have further accelerated the development of nonclinical bioprinted models by supporting in vitro and in silico methods alongside traditional animal testing [16]. These bioengineered systems now enable more accurate evaluation of drug effects on reproductive tissues, addressing longstanding gaps in maternal and fetal health research.

This review offers a detailed overview of recent progress in 3D bioprinting for female reproductive tissue engineering, with a focus on key organs such as the ovary, endometrium, cervix, andvagina. It discusses organ-specific bioink development, bioprinting techniques, and the functional restoration of reproductive tissues. In addition, the review addresses persistent challenges including vascularization, innervation, and barriers to clinical translation, while also exploring future directions involving artificial intelligence (AI) and multi-organ platforms. By integrating these perspectives, the review emphasizes the potential of 3D bioprinting to advance reproductive health research and personalized therapeutic strategies.

## 2 Concept and key technologies of bioprinting

**Fig. 1:**
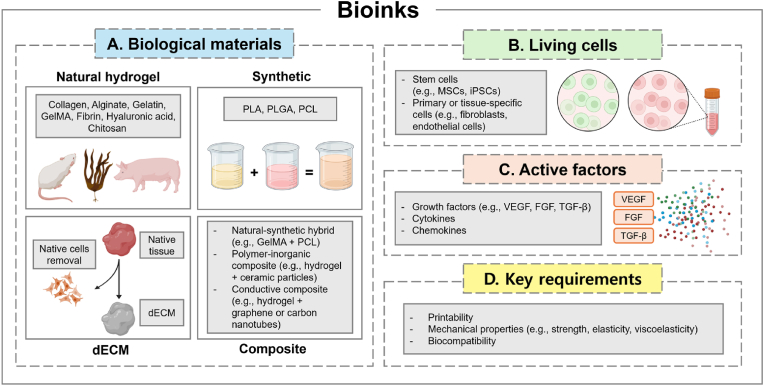
Essential components of bioinks for 3D bioprinting in reproductive tissue engineering. (A) *Biological materials* comprise natural hydrogels (e.g., collagen, alginate, gelatin, GelMA, fibrin, hyaluronic acid), synthetic polymers (e.g., PLA, PLGA, PCL), decellularized extracellular matrix (dECM) derived from native tissues, and composite formulations that integrate natural-synthetic hybrids or functional additives (e.g., ceramics, carbon nanotubes) to enhance structural and biological performance (B) *Living cells*, including stem cells (e.g., MSCs, iPSCs) and primary or tissue-specific cells (e.g., fibroblasts, endothelial cells), are incorporated to restore tissue-specific functions and support regeneration. (C) *Active factors* such as growth factors (e.g., VEGF, FGF, TGF-β), cytokines, and chemokines guide cell behavior within the bioprinted constructs, influencing proliferation, migration, and differentiation. (D) *Key requirements* for bioinks include printability, tunable mechanical properties (e.g., strength, elasticity, viscoelasticity), and biocompatibility to ensure structural fidelity and tissue maturation.

Bioprinting combines 3D printing principles with biological complexities to fabricate tissues and organs layer by layer using bioinks. Bioinks are central to this process, comprising living cells, ECM components, growth factors, and biocompatible materials that support the creation of functional tissue constructs [17,18] (Fig. 1).

The primary role of bioinks is to maintain cell viability and functionality during and after printing. To achieve this, bioinks must exhibit specific rheological properties for smooth extrusion or droplet formation while remaining biocompatible to support encapsulated cells [19]. Additionally, bioinks should promote cell adhesion, proliferation, and differentiation for functional tissue formation [20]. Natural polymers, such as collagen, gelatin, fibrin, and hyaluronic acid (HA), are widely used for their ECM-mimicking properties, while synthetic polymers like polyethylene glycol (PEG) and poly lactic-co-glycolic acid (PLGA) offer tunable mechanical properties. Composite bioinks which combine natural and synthetic materials, provide tailored properties for diverse applications, enhancing the versatility of bioprinted tissues.

Decellularized ECM (dECM) bioinks represent a major advancement in the field [21] (Fig. 1). Derived from natural tissues, dECM bioinks retain critical biochemical cues, structural proteins, and growth factors essential for tissue-specific functionality [22]. These bioinks promote superior cell attachment, proliferation, and differentiation compared to synthetic alternatives, making them highly suitable for regenerative medicine and tissue engineering applications [23].

**Fig. 2:**
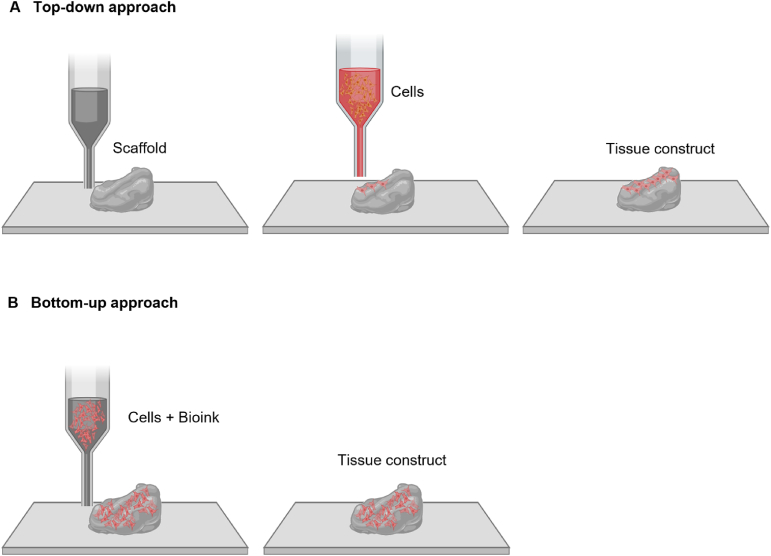
A schematic diagram of a top-down and a bottom-up approach. (A) In the top-down approach, a porous scaffold is first printed, onto which cells are seeded. As the cells proliferate and migrate within the scaffold, they eventually form a tissue construct that mimic native tissue. (B) In the bottom-up approach, cells are directly incorporated into bioink and printed layer by layer to form tissue constructs, allowing for more precise spatial organization of cells and biomaterials. This method eliminates the need for pre-formed scaffolds.

Bioprinting employs two principal strategies: the top-down and bottom-up approaches (Fig. 2). In the top-down method, scaffolds are first fabricated and then seeded with cells, mimicking the tissue's architecture [24]. While effective, this approach often faces challenges with vascularization and nutrient diffusion in thicker tissues. Conversely, the bottom-up approach constructs tissues by assembling modular units, such as spheroids or microtissues [25], offering better control over the microenvironment and enabling natural tissue formation [26]. These methods are particularly advantageous for engineering complex structures like microvessels and fibers [27].

**Fig. 3:**
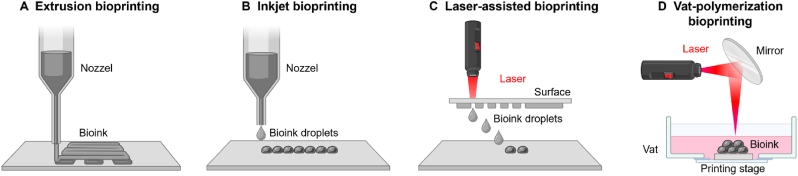
Representative 3D bioprinting modalities. (A) *Extrusion bioprinting* delivers continuous strands of bioink through a nozzle to build structures layer by layer. (B) *Inkjet bioprinting* forms patterns using bioink droplets ejected in a controlled manner. (C) *Laser-assisted bioprinting* employs laser energy to deposit cell-laden droplets without direct contact. (D) *Vat-polymerization bioprinting* utilizes light-mediated curing of bioresins within a vat to fabricate high-resolution constructs.

The core principle of bioprinting involves the layer-by-layer deposition of bioinks to fabricate tissue structures based on digital designs [28]. Among the primary techniques, extrusion bioprinting extrudes continuous filaments of bioink to build volumetric structures, making it suitable for high-viscosity materials [29]. Inkjet bioprinting generates bioink droplets through thermal or piezoelectric actuation, enabling rapid and precise patterning. Laser-assisted bioprinting employs focused laser pulses to transfer bioink droplets onto a substrate with high spatial accuracy and without direct contact [30]. Vat-polymerization bioprinting, encompassing stereolithography (SLA) and digital light processing (DLP), utilizes light-induced polymerization within a bioink-filled vat to achieve ultrafine resolution and complex geometries [31]. The selection of bioprinting modality depends on specific requirements such as printing resolution, bioink rheology, cell viability, and structural complexity (Fig. 3).

Post-processing plays a crucial role in ensuring bioprinted constructs achieve the desired biological and mechanical properties. Printed tissues are typically incubated in bioreactors that provide optimal conditions for cell proliferation, differentiation, and ECM formation [32]. Growth factors and signaling molecules can be added to guide tissue maturation, while mechanical conditioning enhances structural integrity and functionality. These steps are essential for preparing bioprinted tissues for clinical or research applications [33].

### 2.1 Types of bioprinting approaches

Bioprinting methods are classified based on the deposition techniques used, with the primary categories being extrusion bioprinting, inkjet bioprinting, laser-assisted bioprinting, and vat-polymerization bioprinting each offering distinct mechanisms and applications (Table 1).

**Table 1:** Comparative summary of key parameters for major bioprinting techniques.

Parameter	Extrusion	Inkjet	Laser-assisted	Vat-polymerization
**Suitable bioink viscosity**	High	Low	Moderate	Low
**Achievable resolution**	≥100 μm	≥50 μm	10–100 μm	≤10 μm
**Cell density**	High	Low	High	Low
**Print speed**	Slow	Fast	Medium	Medium
**Cell viability**	≤90 %	≥85 %	≥95 %	≥90 %
**Relative equipment cost**	Medium	Low	High	High
**Reference**	[[42], [43], [44]]	[43,45,46]	[43,47,48]	[40,41,49]

Extrusion bioprinting utilizes the continuous deposition of bioink through a nozzle, forming a filament that is layered to create constructs [34]. This technique is highly versatile, accommodating a broad range of bioink viscosities and supporting the production of larger tissue constructs. Its scalability makes it particularly suitable for fabricating sizable constructs, such as ovarian or uterine models, which require structural integrity. However, extrusion bioprinting often provides lower resolution compared to other techniques and may subject cells to shear stress, which can negatively impact cell viability [35].

Inkjet bioprinting, inspired by traditional inkjet printing, operates by ejecting small droplets of bioink through a nozzle onto a substrate [36]. It is characterized by high resolution and gentle handling of cells, ensuring good cell viability. These features make it ideal for creating delicate structures such as endometrial layers or intricate vascular networks needed for studies of implantation and angiogenesis. Despite these advantages, inkjet bioprinting is limited by susceptibility to nozzle clogging and the restricted range of bioink viscosities it can accommodate, which may constrain material choices [37].

Laser-assisted bioprinting employs a laser to transfer bioink from a donor slide to a receiving substrate with precision [38]. This technique is notable for its high resolution and ability to construct intricate structures, making it particularly valuable for modeling reproductive tissues that require precise cellular organization, such as the ovarian follicle or cervical epithelium. However, the high cost and technical complexity of the equipment present significant barriers to its widespread adoption [39].

Vat-polymerization bioprinting uses light-based technologies such as SLA or DLP to solidify photopolymerizable bioinks with exceptional precision. It offers ultrahigh resolution (often <10 μm) and high cell viability, making it well-suited for modeling fine reproductive structures like the endometrium [40]. However, its high cost, material limitations, and lower cell density capacity due to photopolymer constraints limit its broader application [41].

In addition to conventional bioprinting approaches, light-based vat-polymerization bioprinting has emerged as a powerful and precise fabrication technology [40]. This method utilizes photoactivatable bioresins that are selectively polymerized using computer-assisted light projection, enabling microscale resolution and structural fidelity. Compared to nozzle-based techniques, vat-polymerization offers superior resolution and supports high cell viability, making it particularly suitable for constructing intricate tissue architectures and accommodating sensitive cell populations, such as immune cells. Its capacity for producing uniform, reproducible constructs underscores its growing utility in regenerative medicine, particularly for generating functional tissues and organoids with complex geometries [50].

Bioprinting has fundamentally transformed tissue engineering by enabling the precise fabrication of complex tissue constructs [51]. The success of these constructions hinges on careful planning to achieve both structural integrity and functional viability. Critical considerations include the selection of appropriate bioinks, the design of scaffold architecture, and the integration of multiple cell types to mimic native tissue environments. The integration of AI with computer-aided design (CAD) tools has further enhanced the precision and efficiency of bioprinting [52]. AI algorithms optimize CAD models for printability, ensuring that intricate designs meet both structural and functional requirements [53]. AI-assisted systems also improve process control and quality assurance, enabling real-time monitoring and adjustments during bioprinting [54]. This combination of AI and CAD facilitates the accurate layer-by-layer deposition of bioinks, allowing researchers to replicate the intricate cellular interactions and microenvironments characteristic of reproductive organs [55]. By incorporating ECM components, growth factors, and hormone-mimicking signals into bioinks, bioprinting not only supports cell viability, proliferation, and differentiation but also advances the development of functional tissue models tailored to reproductive health research and personalized therapies.

### 2.2 Organ-specific bioinks for female reproductive tissue engineering

The development of bioinks has become a critical aspect of 3D bioprinting for female reproductive system engineering, providing the necessary biochemical and biophysical cues to support cell survival, differentiation, and tissue regeneration. Female reproductive organs such as the ovary, endometrium, vagina, cervix, and placenta exhibit unique biomechanical properties and cellular compositions, requiring tailored bioink formulations to recreate their microenvironments effectively. Traditional treatments for premature ovarian insufficiency (POI), intrauterine adhesion (IUA), vaginal agenesis, cervical cancer, and pregnancy-related disorders often face challenges such as donor shortages, immune rejection, and limited regenerative capacity. As an emerging solution, 3D bioprinting combined with bioengineered materials has demonstrated remarkable potential in disease modeling, transplantation, and reproductive tissue regeneration (Table 2).

**Table 2:** Bioprinting parameters and applications for female reproductive organ models.

Organ	Target disease	Bioprinting technique	Category	Material (Bioink type)	Cell type	Application	Ref
Ovary		Extrusion	Natural	GelMA (10 % w/v) + Alginate (5 % w/v)	Mouse granulosa cell	3D printed scaffold for ovarian follicles	[73]
		Extrusion	Natural	Porcine ovary dECM + Gelatin (15 % w/v) + Sodium Alginate (3 % w/v)	Mouse primary ovarian cell (POC)	3D bioprinted scaffold for ovarian failure	[57]
	Premature Ovarian Insufficiency (POI)	Extrusion	Natural	Gelatin (10 % w/v)	Murine follicles	3D printed scaffold for transplantation	[74]
	Infertility	Electrospinning	Synthetic	Polycaprolactone (PCL)	Preantral (PA) follicles	3D-printed scaffold for multiple PA follicle development	[75]
	Ovarian cancer	Extrusion	Natural	Sodium Alginate (2 % w/v) + Gelatin (15 % w/v)	SKOV-3 ovarian cancer cell + MeWo cancer fibroblasts	3D bioprinted ovarian tumor model	[76]
		Two-photon polymerizations	Synthetic	ORMOCER (urethane/alkoxysilane hybrid polymer) + SU-8	GFSHR-17 rat granulosa cells	ORMOCER (Photosensitive organic–inorganic hybrid polymer)	[77]
		Extrusion	Natural	Gelatin (10 % w/v)	Secondary follicles	3D printed microporous hydrogel scaffolds	[78]
		Extrusion bioprinter	Natural	GelMA (8 % w/v)	Human ovarian granulosa tumor cell line (COV434, KGN), Murine ovarian surface epithelial cells (EECs) (ID 8), Murine ovarian somatic cells	Cell-laden 3D printing of artificial ovaries	[65]
Endometrium		Extrusion	Natural	Alginate (2 % w/v) + Hyaluronic acid (0.5 % w/v)	Neonatal rat endometrial stromal cells (ESCs), EEC	3D bioprinted endometrium model	[58]
	Endometrial injury	Extrusion	Natural	Sodium Alginate (4 % w/v) + Gelatin (20 % w/v)	human induced mesenchymal stem cell (hiMSC)	3D bioprinted hiMSC-loaded hydrogel scaffold for repair of damaged uterine endometrium	[79]
	Intrauterine adhesion (IUA)	Selective laser sintering	Synthetic	Poly lactic-co-glycolic acid (PLGA)	none	3D printed scaffold endometrium model	[80]
	IUA	Powder filament extruder	Synthetic	Degradable polymeric film (DPF)	none	3D printed film for endometrium	[81]
	IUA	Extrusion	Synthetic, Natural	Gelatin/Alginate hydrogel + PLGA microspheres	Rat ESCs	3D printed hydrogel scaffold for sustained G-CSF delivery and endometrial regeneration	[61]
		Extrusion	Natural	GelMA + Collagen methacryloyl (ColMA)	Amniotic Mesenchymal stem cells (AMSCs)	Bioprinting of a blue light-cross-linked biodegradable hydrogel encapsulating amniotic MSCs	[71]
Pelvic Floor (uterus, vagina)	Pelvic Organ Prolapse (POP)	Extrusion	Natural	Aloe Vera (1 % w/v) + Sodium Alginate (1 % w/v)	Human endometrial mesenchymal stem cell (MSC)	3D bioprinted eMSC-laden hydrogel on melt electrospun PCL mesh for pelvic floor repair	[82]
Myometrium		Magnetic levitation		Myometrial 3D cell rings	human uterus smooth muscle cell (HUtSMC)	3D bioprinted uterine rings	[83]
Vagina	Rat vaginal reconstruction models	Stereolithography (SLA)-based 3D printer	Synthetic, Natural	dECM hydrogel + GelMA (8 % w/v) + Silk fibroin (0.3g) + Photoinitiator (0.5 %)	hUCMSCs (passage3)	3D printing of the lumen scaffold	[70]
	Vaginal agenesis	Extrusion 3D printer (FDM)	Synthetic	PLA + Oxidized regenerated cellulose	–	3D printed PACIENA prosthesis for vaginoplasties	[84]
	Vaginal agenesis	Extrusion	Natural	Porcine vagina dECM + Gelatin + Alginate	human Bone Marrow-derived Mesenchymal Stem Cells (BM-MSC)	3D vagina tissue printed with extracellular matrix (ECM) bioink	[64]
	Vaginal reconstruction	Extrusion	Natural	GelMA (8 % w/v) + Photoinitiator (5 %) + Vascular ECM + Silk Fibrin (5 % w/v)	Bone marrow stem cells (BMSCs), HUVEC	3D bioprinted scaffold	[69]
Cervix	Cervical tumor	Extrusion	Natural	Alginate (2.5 % w/v) + Mannitol (3.3 % w/v) + CaCl_2_ (0.19 % w/v)	HeLa cells (CCL-2)	Cervical tumor model obtained by 3D bioprinting of HeLa cells in an Alg-based matrix	[85]
	Cervical cancer	Low-temperature deposition manufacturing (LDM) extrusion	Synthetic	Biocompatible polyurethane	HUVECs, HeLa cells	Personalized 3D-printed cervical tissue implant with anti-HPV protein release function	[59]
	Cervical atresia	Extrusion 3D printer	Synthetic	Silk Fibrin/PCL + Hexafluoroisopropanol (HFIP)	human cervical squamous epithelial (hCSECs), human cervical columnar epithelial cells (hCCECs), human ESCs (hESCs)	Hybrid stent by combining electrospinning and 3D printing	[86]
Placenta	Preeclampsia (PE)	Extrusion	Natural	GelMA	BeWo cells (trophoblast cell) hMSC	3D-printed, bioengineered placenta model	[87]
		Extrusion	Natural	GelMA	Trophoblast HUVEC	Dynamic bioprinted placenta model	[63]
Implantation		Extrusion	Synthetic	Polydimethylsiloxane (PDMS)	None	Implantation model	[60]

Bioinks for reproductive tissue bioprinting can be broadly categorized into natural, synthetic, and hybrid biomaterials, each offering distinct advantages in terms of biocompatibility, mechanical properties, and bioactivity. Natural bioinks, such as gelatin, alginate (Alg), collagen, gelatin methacryloyl (GelMA), fibrin, HA, silk fibroin (SF), and dECM, provide excellent biocompatibility and biofunctionality, closely mimicking the ECM of native tissues. For instance, gelatin-Alg hydrogels have been extensively utilized in ovarian tissue engineering, where they support follicle growth and hormone production, facilitating fertility restoration [56]. Additionally, porcine ovarian dECM combined with sodium Alg and gelatin has been used to construct bioprinted ovarian scaffolds, offering a biochemically rich microenvironment that promotes granulosa cell proliferation and follicular development [57]. Similarly, endometrial regeneration has been explored using HA-Alg hydrogels, which enhance endometrial epithelial and stromal cell adhesion, improving implantation efficiency and tissue repair in patients with uterine damage [58]. While natural bioinks excel in bioactivity, they often lack mechanical strength and structural stability, necessitating the integration of synthetic polymers. Polycaprolactone (PCL),PLGA, polydimethylsiloxane (PDMS), and polyurethane (PU) are commonly used synthetic bioinks that provide mechanical reinforcement and allow for the fabrication of long-lasting, load-bearing reproductive tissue scaffolds. For example, PCL-based scaffolds have been implemented for cervical and vaginal tissue engineering, where low-temperature deposition manufacturing (LDM) techniques enable the construction of hierarchically porous structures that facilitate cell migration and vascularization [59]. Similarly, PDMS scaffolds have been employed in endometrial and embryo implantation studies, where their tunable porosity and elasticity allow for the replication of uterine microenvironments [60]. In recent years, hybrid bioinks that integrate natural and synthetic components have been developed to balance bioactivity with mechanical stability, thereby improving long-term functional outcomes. For instance, protein-dextran–PLGA composites have been successfully used to fabricate hydrogel scaffolds enriched with granulocyte colony-stimulating factor (G-CSF), which promotes endometrial regeneration and improves pregnancy outcomes [61]. In placental bioprinting, GelMA-based bioinks containing trophoblast cells and endothelial cells have enabled the creation of dynamic placenta models, providing new platforms for studying trophoblast invasion, maternal-fetal interactions, and preeclampsia [62,63]. Furthermore, vaginal reconstruction efforts have incorporated bioprinted porcine vaginal dECM combined with gelatin and Alg, resulting in functional, biocompatible vaginal tissue models that can be transplanted for vaginal agenesis repair [64].

**Fig. 4:**
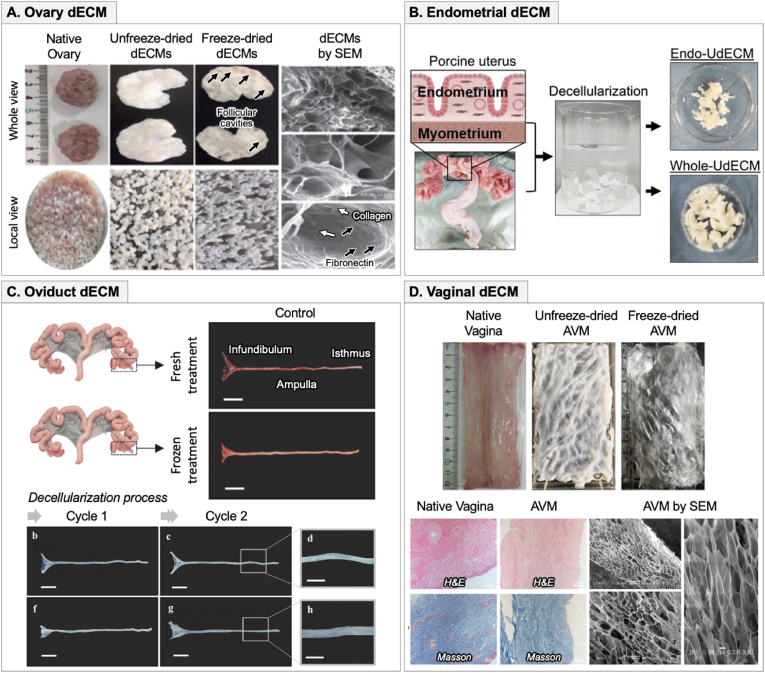
ECM-derived bioinks for reproductive tissue bioprinting. (A) Ovary dECM bioink maintains follicular structure and supports hormone secretion. Representative images show native ovary, unfreeze-dried and freeze-dried dECMs, and scanning electron microscopy (SEM) visualization highlighting ECM components such as collagen and fibronectin. Reproduced with permission from Ref. [57], ©2022 International Journal of Bioprinting. (B) Endometrial dECM bioink derived from porcine uterus supports endometrial regeneration. The schematic illustrates the decellularization process, with comparisons between Endo-UdECM and Whole-UdECM. Reproduced with permission from Ref. [66], ©2023 Advanced Functional Materials. (C) Oviduct dECM bioink retains structural integrity for embryo transport. The decellularization process is depicted alongside control and treated samples, demonstrating preserved morphology across different treatments. Reproduced with permission from Ref. [68], ©2025 Theriogenology. (D) Vaginal dECM bioink enhances epithelial remodeling and structural integrity. Images compare native vaginal tissue with unfreeze-dried and freeze-dried AVM bioinks. Histological staining (H&E, Masson) and SEM images confirm ECM preservation and microstructural characteristics. Reproduced with permission from Ref. [64], ©2021 International journal of biological macromolecules.

The ovary is a highly specialized organ responsible for follicular development, hormone production, and oocyte maturation, necessitating bioinks that can mimic its dynamic microenvironment. To address this challenge, ovary-derived dECM bioinks have been developed to replicate the follicular niche, providing a supportive biochemical and structural framework for follicle survival. Zheng et al. formulated an ovarian dECM bioink, which successfully preserved hormone secretion and follicular viability in a mouse model of ovarian failure, demonstrating its potential for fertility restoration [57] (Fig. 4A). Similarly, Wu et al. demonstrated that GelMA-based bioinks provided a biomechanically stable scaffold for artificial ovary bioprinting, significantly enhancing follicular survival and maintaining structural integrity [65]. These advances suggest that bioprinted ovarian constructs could be instrumental in treating ovarian insufficiency and improving in vitro follicle maturation systems.

The endometrium undergoes cyclic remodeling in response to hormonal cues, requiring bioinks that preserve its dynamic regenerative capacity. Endometrial dECM-based bioinks offer an organ-specific biochemical environment, supporting epithelial-stromal interactions essential for implantation and tissue repair. Ahn et al. developed uterus-derived dECM (UdECM) hydrogels, demonstrating their ability to enhance endometrial regeneration and restore fertility by replicating the ECM composition of distinct uterine layers (Fig. 4B) [66]. Furthermore, Nie et al. engineered a bilayer endometrial construct (EC) using a sodium Alg-HA bioink, successfully restoring full-thickness endometrial function in a rat model [58]. In another approach, Wen et al. designed a G-CSF-loaded hydrogel scaffold, which promoted vascularization and epithelial regeneration in IUA models [61]. These findings highlight the potential of bioprinted ECs to serve as therapeutic scaffolds for patients with endometrial dysfunction and infertility.

The oviduct (fallopian tube) is a critical structure for fertilization and embryo transport, necessitating bioinks that can mimic its ciliated epithelial structure and peristaltic function. Oviduct-derived dECM bioinks have been explored to replicate its physiological microenvironment, ensuring proper embryo development and transport. Koo et al. developed a 3D-bioprinted GelMA hydrogel culture system, successfully recreating key oviduct ECM properties, which significantly enhanced preimplantation embryo development [67]. Similarly, Martínez-López et al. employed decellularized porcine oviduct scaffolds, demonstrating high structural retention and functional support for spermatozoa and early embryos (Fig. 4C) [68]. These advancements pave the way for future bioprinted oviduct models that could be applied in reproductive toxicology studies, assisted reproductive technology, and fallopian tube regeneration.

Bioprinting approaches for vaginal reconstruction require bioinks that promote epithelialization, mechanical resilience, and immune compatibility. Vaginal dECM-derived bioinks have been developed to support structural and functional regeneration, offering promising solutions for vaginal agenesis and post-surgical reconstruction. Hou et al. developed a biomimetic 3D vaginal tissue model using an acellular vaginal matrix (AVM) bioink, which successfully supported vaginal tissue reconstruction with functional epithelial layer formation (Fig. 4D) [64]. Additionally, Zheng et al. designed a composite bioink, integrating vaginal-derived ECM, GelMA, and SF, which enhanced epithelial adhesion and tissue remodeling, indicating its potential for improving vaginal graft integration [69]. Shi et al. further optimized a SLA-printed dECM/MSCs-exosome bioink, demonstrating accelerated neovascularization and epithelial repair in vaginal reconstruction models [70]. These findings highlight the clinical potential of dECM-based vaginal bioinks in personalized regenerative therapies for vaginal defects and reconstructive surgeries.

The uterus presents unique challenges for bioprinting due to its multilayered structure, requiring bioinks that recapitulate both myometrial and endometrial properties. Recent studies have explored UdECM bioinks capable of supporting uterine tissue regeneration and functional remodeling. Feng et al. engineered a blue-light-crosslinked hydrogel encapsulating amniotic mesenchymal stem cells (AMSCs), which reduced fibrosis and improved endometrial thickness in IUA models [71]. Furthermore, Chen et al. developed a biomimetic tri-layer scaffold with controlled E2 release, effectively supporting uterine tissue regeneration and hormonal regulation [72]. These advancements in uterine bioprinting suggest that dECM-based bioinks could revolutionize treatments for uterine disorders, offering bioengineered solutions for conditions such as Asherman's syndrome and uterine factor infertility.

The integration of dECM-based bioinks with 3D bioprinting technologies has significantly advanced female reproductive tissue engineering, offering customized, bioactive scaffolds for ovarian, endometrial, oviductal, vaginal, and uterine regeneration. These organ-specific bioinks not only replicate tissue-specific ECM compositions but also support cellular adhesion, proliferation, and differentiation, essential for functional tissue regeneration. Despite these advancements, several challenges remain in the development of bioinks for female reproductive tissue engineering. One of the major limitations is the lack of vascularization in bioprinted constructs, which restricts nutrient diffusion and long-term tissue viability. To address this, researchers are exploring bioinks embedded with pro-angiogenic factors, endothelial cells, and controlled-release growth factors to promote vascular network formation within bioprinted reproductive tissues. Additionally, shear stress during extrusion bioprinting can compromise cell viability, necessitating the development of shear-thinning bioinks with enhanced printability and cell encapsulation properties.

## 3 3D bioprinting applications in the female reproductive system

The development of physiologically relevant in vitro models for female reproductive tissues requires precise replication of hormone responsiveness, multicellular architecture, and microenvironmental cues. 3D bioprinting provides a robust platform for spatially organizing cells and biomaterials to emulate the structural and functional features of native tissues. For example, bioprinted endometrial constructs have been designed to mimic cyclical remodeling, while engineered ovarian tissues can recapitulate follicular arrangement essential for hormone production and oocyte maturation [7,[88], [89], [90]]. These models serve as valuable tools for studying reproductive processes, evaluating infertility treatments, and developing regenerative therapies for conditions such as endometrial damage and ovarian dysfunction.

**Fig. 5:**
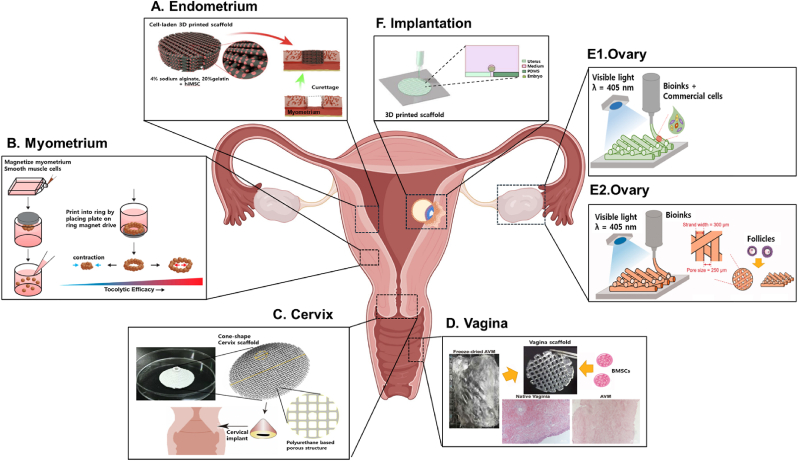
Schematic overview of 3D bioprinting applications in modeling female reproductive tissues. (A) Bioprinted endometrial constructs incorporating hMSCs within cell-laden hydrogels, designed for in vitro culture and in vivo transplantation to support cyclic regeneration [79]. © 2020 Acta biomaterialia. (B) Myometrial model using magnetically responsive smooth muscle cell-laden constructs to assess contractility and tocolytic drug efficacy. Reproduced from Ref. [83] under an open-access license, © 2017 International Journal of molecular sciences. (C) Cervical reconstruction using 3D-printed polyurethane (PU) scaffolds with tailored porosity and elasticity to match native cervical tissue biomechanics [59]. © 2020 Biomedical materials. (D) Vaginal tissue constructs printed with acellular vaginal matrix (AVM) bioinks and BMSCs, supporting epithelial regeneration and tissue integration [64]. © 2021 International journal of biological macromolecules. (E1) Ovarian scaffold bioprinted with GelMA and commercial ovarian cell lines (COV434, KGN, ID8) to replicate hormone production and stromal structure. (E2) Follicle-laden ovarian scaffold using visible light–crosslinked GelMA bioinks for follicular development and oogenesis. Reproduced from Ref. [65] under an open-access license, © 2021 Climacteric. (F) Implantation platform based on 3D-printed scaffolds for visualizing embryo adhesion and modeling early implantation events in vitro. Reproduced with permission from Ref. [60], © 2022 Journal of cellular physiology.

Recent advances have led to the development of increasingly specialized bioprinted constructs that capture the physiological complexity of distinct reproductive tissues. As illustrated in Fig. 5, various strategies have been employed to model endometrial regeneration using cell-laden scaffolds (Fig. 5A), simulate myometrial contractility through magnetically responsive smooth muscle constructs (Fig. 5B), and reconstruct cervix-like implants with tailored mechanical properties (Fig. 5C). Vaginal tissue models incorporating dECM bioinks and stem cells have demonstrated potential for epithelial regeneration and integration (Fig. 5D), while ovarian constructs using GelMA-based scaffolds or commercial tumor cell lines replicate follicular development and hormone activity (Fig. 5E1–E2). Furthermore, bioprinted systems that support embryo implantation and early developmental stages offer new tools for studying implantation mechanisms (Fig. 5F).

Each of these models integrates cell type–specific bioinks, optimized scaffold geometries, and bioprinting modalities tailored to the unique physiological demands of each tissue. These platforms not only capture essential features such as hormone responsiveness, cyclic remodeling, and mechanical compliance but also support applications in disease modeling, drug screening, and tissue repair. The following sections provide a detailed overview of tissue-specific advances in female reproductive bioprinting.

### 3.1 Endometrium reconstruction

The endometrium is one of the most dynamic tissues in the human body, with its thickness varying significantly in response to physiological conditions. It exhibits a high degree of plasticity, responding to endocrine signals by proliferating in preparation for embryo implantation or undergoing cyclical shedding to initiate a new menstrual phase [91]. Disruptions in its function or structural integrity can lead to conditions such as menorrhagia [92]. The smooth muscle layers of the uterus (myometrium) facilitate the expulsion of menstrual effluent while maintaining the structural integrity of the uterine contents [93]. Pharmacological interventions can modulate endometrial thickness at different points in the menstrual cycle, influencing vascularization and the proliferation of pathological structures, thereby altering the size and extent of anatomical abnormalities [94,95].

Events such as curettage or inflammation can damage the endometrium, leading to fibrosis and adhesion within the uterine cavity, which can result in implantation failure [96]. Researchers have extensively investigated bioengineered models of the uterus, utilizing various biomaterials such as endometrial cells, collagen, matrigel, silk, and other biodegradable polymers [90]. These models are typically developed and studied under controlled laboratory conditions to simulate uterine structure and function for research purposes.

**Fig. 6:**
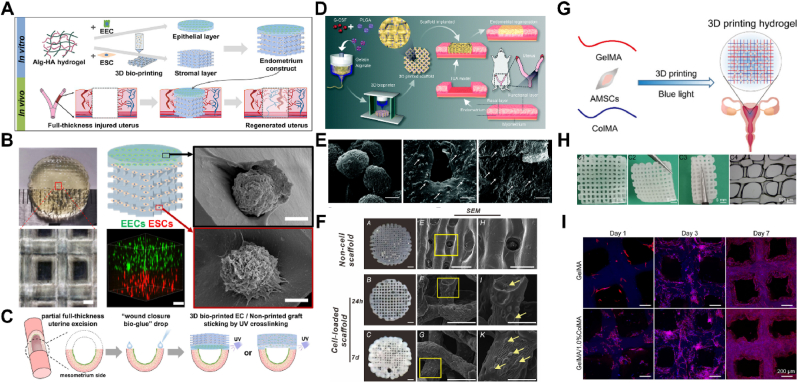
3D printing of the endometrium. (A) A graphical abstract illustrating the 3D printing of an endometrial construct (EC) and its application in vivo. (B) Characterization of the printed layers using bright-field and SEM imaging. (C) A schematic diagram showing the partial full-thickness uterine excision and the implantation procedures for either a 3D bioprinted EC or a non-printed graft. Reproduced with permission from Ref. [58] ©2023 Acta biomaterialia. (D) Overview of the experimental design for a 3D-printed granulate colony-stimulating factor-loaded sustained-release microsphere system. (E) Structure and morphology of the microsphere-scaffold visualized under SEM. Reproduced with permission from Ref. [61], ©2022 Biomaterials science. (F) Characterization of the 3D printed hydrogel scaffold loaded with hiMSCs, as seen under SEM. Reproduced with permission from Ref. [79], © 2020 Acta biomaterialia. (G) A schematic diagram illustrating the construction of 3D printed hydrogel loaded with cells. (H) Macroscopic view of the printed porous hydrogel. (I) Immunofluorescence images showing human amnion mesenchymal stem cells in the printed scaffolds. Reproduced with permission from Ref. [71], ©2021 ACS omega.

To mimic the endometrial structure, a bilayer cell-loaded EC was developed using 3D extrusion-based bioprinting. This construct includes a dense epithelial layer and a porous, grid-like stromal layer composed of sodium Alg-HA hydrogel for endometrial regeneration. The upper layer contains endometrial epithelial cells (EECs), while the lower layer is embedded with endometrial stromal cells (Fig. 6A and B). In a rat model, the 3D bioprinted EC successfully repaired severely damaged endometrium, promoting regeneration of the endometrium, glands, myometrium, and angiogenesis (Fig. 6C). Notably, it restored fertility in injured uteri, indicating its potential as a therapeutic option for women with damaged endometrium and infertility [58].

Furthermore, a 3D printed hydrogel combined with a sustained-release microsphere (SRM) system was developed to create a 3D printed G-CSF-SRM system in vitro. This system allowed precise spatial control of drug distribution and structural individualization, increasing local G-CSF concentration with sustained release (Fig. 6D). In a Sprague-Dawley rat model of IUAs, the 3D microspheres promoted endometrial regeneration, reduced fibrosis, and improved epithelial, stromal, and vascular regeneration. Using 3D extrusion-based bioprinting, a bilayer EC with sodium Alg-HA hydrogel was fabricated, restoring endometrial structure and function (Fig. 6E). In a rat model, the EC improved tissue regeneration and reproductive outcomes post-implantation, demonstrating potential for treating severe endometrial injuries [61].

Similarly, to address endometrial injury resulting from uterine curettage, a porous, grid-like hydrogel scaffold loaded with human induced mesenchymal stem cells (hiMSCs) was fabricated using 3D bioprinting technology (Fig. 6F) [79]. This 3D-printed hydrogel scaffold created a supportive in vitro microenvironment for hiMSCs, significantly enhancing the survival of transplanted hiMSCs compared to direct local administration of hiMSCs in vivo. hiMSC transplantation triggers early host immune responses, with serological effects lasting over a month and local responses for about a week. Although the regenerated endometrium did not fully restore normal structure and function, the 3D-printed hiMSC-loaded scaffold improved endometrial histomorphology, including tissue, gland, stromal, epithelial, and endothelial cell regeneration, and enhanced functional indicators of endometrial receptivity.

IUA is a common complication following uterine surgery, often leading to clinical symptoms such as reduced menstrual flow, amenorrhea, recurring lower abdominal pain, and infertility. A composite hydrogel made of GelMA/ColMA has been developed using 3D printing technology to prevent IUA (Fig. 6G) [71]. This anti-adhesion hydrogel features a porous 3D structure, enhanced mechanical strength, and extended degradability (Fig. 6H). In vitro experiments demonstrated that stem cells could successfully attach and evenly spread along the inner wall channels, exhibiting high survival rates, normal proliferation, and healthy cell morphology (Fig. 6I). The GelMA/ColMA/human amnion mesenchymal stem cell (MSC) hydrogel has been shown to effectively prevent IUA in a rat IUA model.

### 3.2 In vitro models for embryo implantation

Understanding embryonic development is a central focus in developmental biology [97]. Embryogenesis provides essential insights into reproductive health and forms the theoretical basis for advancements in tissue-engineered organ reconstruction [98,99]. Due to the limitations of studying embryo development in vivo, current research primarily concentrates on the preimplantation stages of mammalian embryogenesis [100]. However, post-implantation studies are limited due to the difficulties associated with direct observation. Therefore, research on post-implantation embryonic development is crucial for advancing our understanding of the development of embryos, tissues, and organs [100].

Currently, the predominant approach for post-implantation embryo culture involves the use of 2D tissue culture plates. Recent advancements in biomaterials have expanded the use of these materials for in vitro post-implantation embryo culture. Examples include tissue culture plates coated with ECM proteins such as collagen, laminin, and fibronectin, as well as co-culturing embryos with uterine endothelial cells or using Matrigel basement membrane matrices [[99], [100], [101]]. Despite these advancements, existing culture techniques often fail to replicate the physiologically relevant microenvironment needed for accurate developmental studies.

**Fig. 7:**
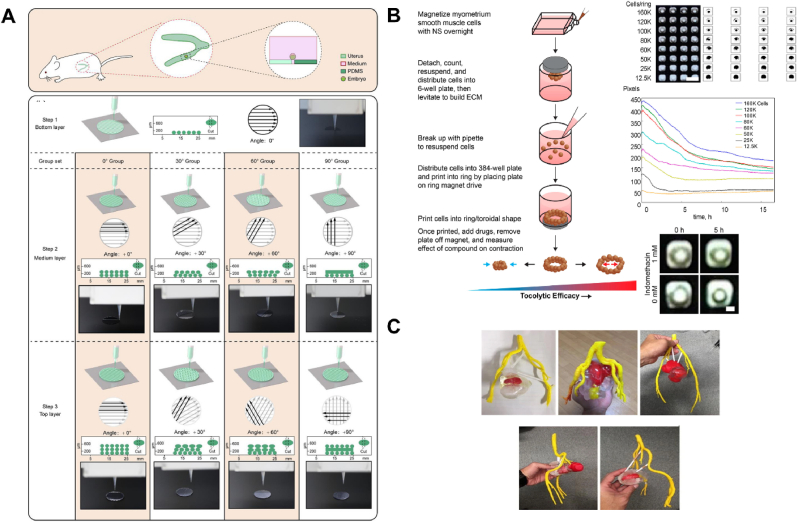
3D printing of embryo implantation and the whole uterus. (A) A schematic illustration of a study where embryos were cultured using controlled contact angles and embryo-scaffold interactions via 3D printing. Reproduced with permission from Ref. [60], © 2022 Journal of cellular physiology. (B) A schematic flow diagram of 3D printed magnetic ring structures used to evaluate a uterine contractility assay. The diagram also shows the analysis of myometrial smooth muscle cells printed at varying densities and their contraction response to indomethacin. Reproduced from Ref. [83] under an open-access license, © 2017 International Journal of molecular sciences. (C) Customized 3D physical models of the uterus created for five different cases of uterine cancer surgical removal. The models depict the uterus (transparent), endometrium (light yellow), endometrial cancer (red), and blood vessels (yellow). Reproduced from Ref. [102] under an open-access license, © 2017 Radiological physics and technology. (For interpretation of the references to colour in this figure legend, the reader is referred to the Web version of this article.)

Recently, 3D printed anisotropic microporous scaffolds, created by adjusting the angle between printed layers, were shown to influence embryo development. Scaffolds with 30° and 60° angles promoted moderate embryo-scaffold attachment, supporting embryo growth (Fig. 7A) [60]. On day 7, T-positive and forkhead box protein A2 (FOXA2) -positive cells were observed migrating from the posterior to the anterior region of the embryo. These findings highlight the role of 3D architecture in embryo implantation and demonstrate that additive manufacturing is a versatile tool for supporting embryonic development in vitro. Successful implantation requires synchronization between the embryo's development and a complex series of molecular and cellular events in the uterus, regulated by paracrine and autocrine signals [103]. During the cleavage stage, the embryo must generate sufficient cells to form the inner cell mass and trophectoderm at the blastocyst stage. The final stage, invasion, begins when syncytiotrophoblasts penetrate the uterine epithelium, followed by mononuclear cytotrophoblasts invading the endometrium, the inner third of the myometrium, and uterine vasculature.

A 3D printed perfusion bioreactor system has been shown to enhance endothelial cell responses by promoting network formation and the expression of angiogenic markers, including Platelet endothelial cell adhesion molecule, matrix metallopeptidase 2, matrix metallopeptidase 9, and vascular endothelial growth factor (VEGF) A [63]. Bioprinting also facilitated the co-localization of trophoblasts and endothelial cells, effectively mimicking in vivo conditions. Further analysis demonstrated that trophoblasts reduced angiogenic responses by limiting network formation, reducing motility, and inducing endothelial cell apoptosis. Additionally, the presence of endothelial cells was found to inhibit trophoblast invasion rates.

### 3.3 Myometrium and whole uterus

The myometrium is the primary layer responsible for uterine contractions, which play crucial roles in various reproductive functions, including menstruation, sperm and embryo transport, pregnancy, and childbirth [104,105]. Abnormalities in uterine contractility can lead to several reproductive disorders, such as preterm labor, premature birth, infertility, abnormal implantation, and irregular menstrual cycles [106]. The 3D bioprinting of myometrial tissue and entire uterine structures holds significant potential for applications in regenerative medicine. Additionally, high-throughput 3D bioprinted tissues offer advanced platforms for drug discovery and toxicology testing, enabling the evaluation of therapeutic compounds and assessment of potential toxic effects [107,108].

Magnetic 3D bioprinting has been employed to organize human myometrial cells into ring structures, which were monitored for contractile activity over time and in response to various clinically relevant agents (Fig. 7B) [83]. Both commercially sourced and patient-derived myometrial cells were bioprinted into rings using a 384-well format for high-throughput analysis of uterine contractility. The bioprinted uterine rings, derived from different cell sources and patients, displayed distinct contractile behaviors and differential responses to uterine contractility inhibitors, such as indomethacin and nifedipine.

An approach has also been proposed to support pre-surgical planning for the uterus by integrating medical image analysis and physical model generation through 3D printing (Fig. 7C) [102]. In this method, patient-specific anatomy and uterine lesions are first segmented from Magnetic resonance (MR) images, and a 3D physical model is then created, replicating the patient's uterus in terms of size and softness, with transparency to allow easy observation of internal structures. In the experiments, pre-surgical hysterectomy models were created for five patients diagnosed with uterine endometrial cancer. An experienced radiologist, surgeons, and all patients participated in subjective evaluations of the model's usefulness. The accuracy of the physical models was quantitatively assessed by comparing the patients' MR images with computed tomography images of the models.

### 3.4 Ovary

The ovary, a paired intraperitoneal endocrine organ located in the lower abdomen, plays a critical role in female reproduction and hormone production. It consists of germ cells, such as oocytes (eggs), and somatic cells, including granulosa cells, thecal cells, and stromal cells [109,110]. The interactions between these cells drive the formation of ovarian follicles, support the development of oocytes and somatic cells. These follicles are essential for ovulation and the subsequent formation of the corpus luteum, a temporary endocrine structure formed after ovulation. The corpus luteum secretes P4, a hormone critical for maintaining the early stages of pregnancy, until the placenta takes over this function [111]. In addition to its role in reproduction, the ovary is responsible for the production of key reproductive hormones, particularly estrogen and P4, which are secreted by granulosa and theca cells. During the proliferative phase of the menstrual cycle, ovarian follicles grow and mature, with one dominant follicle being selected for ovulation. A surge in LH triggers the release of the oocyte, which then travels through the fallopian tubes toward the uterus. [112]. If fertilization occurs, the blastocyst secretes human chorionic gonadotropin, signaling the corpus luteum to continue producing P4, which is crucial for sustaining pregnancy until the placenta can assume this role [113]. If fertilization does not occur, the corpus luteum degenerates into the corpus albicans, resulting in a drop in P4 levels, which leads to the onset of menstruation. Disruptions in these ovarian feedback loops, whether due to hormonal imbalances, ovarian dysfunction, or structural anomalies, can result in infertility, chronic pain, or reproductive hormonal disorders [114].

**Fig. 8:**
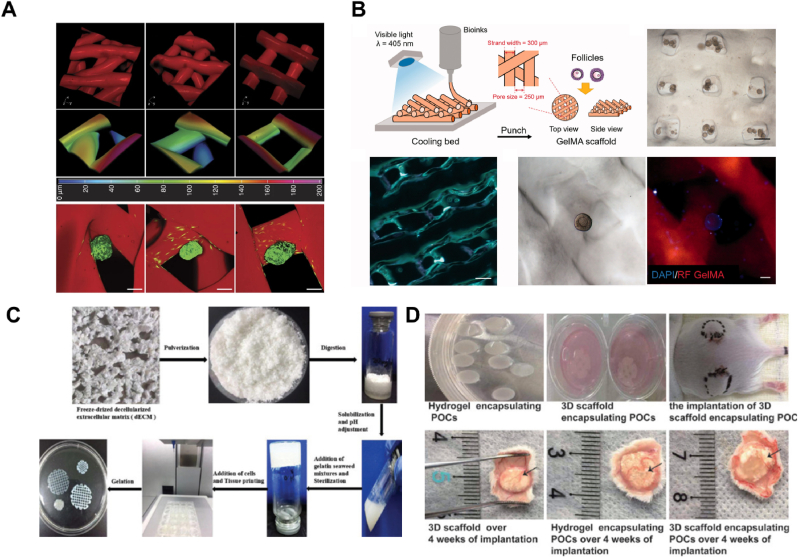
3D printing of the ovary. (A) 3D reconstructions from confocal fluorescence image stacks of 3D printed scaffolds, visualized from various angles to show structural complexity. Reproduced from Ref. [65] under an open-access license, © 2017 Nature communications [78]. (B) A schematic representation of the 3D bioprinting process for ovarian follicles, along with bright-field and confocal fluorescence images of the 3D printed follicular constructs. The cooling bed is used to support the formation of the porous GelMA scaffold. Reproduced from Ref. [65] under an open-access license, © 2021 Climacteric. (C) The biofabrication process of bioink derived from ovarian dECM, followed by the 3D printing of porous scaffold structures. (D) Comparative images of 3D printed scaffolds encapsulating POCs before and after implantation, showing tissue integration and scaffold degradation over several weeks post-implantation. Reproduced from Ref. [57] under an open-access license, © 2022 International journal of bioprinting.

Recent advancements in 3D bioprinting have explored ovarian function restoration. For example, 3D-printed microporous hydrogel scaffolds have been used to investigate how pore geometry affects ovarian follicle survival. Scaffolds were printed at different angles, 30°, 60°, and 90° to assess their impact on follicle interaction and viability. Scaffolds with 30° and 60° angles created more interaction between the scaffolds and follicles, which enhanced follicle survival, while 90° scaffolds, with more open porosity, limited this interaction (Fig. 8A) [78]. When implanted into sterilized mice, these follicle-seeded scaffolds became vascularized, fully restoring ovarian function, with healthy pups being born via natural mating. Additionally, a 3D bioprinted ovary was constructed using GelMA bioink, demonstrating the ability to support follicle structure, growth, and maturation in vitro (Fig. 8B) [65]. In another study, cell-laden 3D printing was applied to create artificial ovaries for the first time using ovarian tumor cell lines and ovarian somatic cells. Despite some initial cell death, these efforts highlight the growing potential of 3D bioprinting in constructing functional ovarian tissue.

For fertility preservation, ovarian dECM has served as a foundational material for reconstituting an artificial ovary (Fig. 8C). This dECM was mixed with a gelatin blend to create a bioink, which was then used to print 3D scaffolds with or without encapsulated primordial ovarian cells (POCs). When implanted into a castrated female mouse model, the 3D scaffolds encapsulating POCs promoted greater cell survival, angiogenesis, and sex hormone secretion compared to controls (Fig. 8D) [57].

### 3.5 Vaginal tissue engineering

**Fig. 9:**
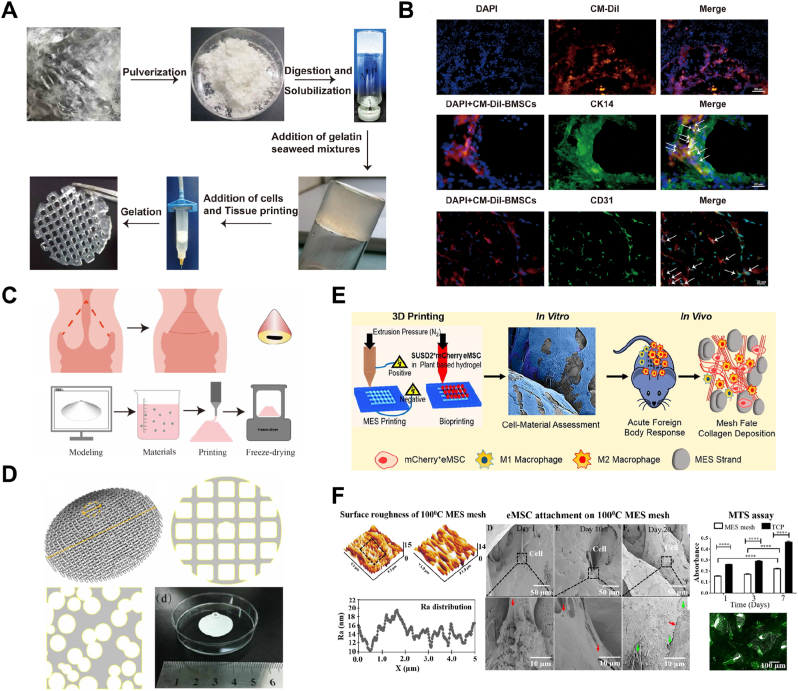
3D printing of vagina and cervix. (A) Production process of bioink using decellularized vaginal tissue and the 3D printing of porous cylindrical scaffold structures. (B) Fluorescence images of 3D printed scaffolds encapsulating CM-Dil-labeled BMSCs. Reproduced with permission from Ref. [64], © 2021 International journal of biological macromolecules. (C) A schematic diagram of the Conization surgery procedure and the corresponding 3D printing system used to reconstruct cervical tissue. (D) 3D printed reconstruction of missing cervical tissue following conization surgery. Reproduced with permission from Ref. [59], © 2020 Biomedical materials. (E) Schematic illustration of bioprinting endometrial mesenchymal stem/stromal cells (eMSC) to improve treatments for pelvic organ prolapse (POP). (F) Characterization of melt electrospun (MES) mesh and assessment of eMSC attachment on the 3D printed mesh, illustrating the mesh's structural properties and cell integration. Reproduced with permission from Ref. [82], © 2019 Acta biomaterialia.

Congenital and acquired conditions, such as Mayer-Rokitansky-Küster-Hauser (MRKH) syndrome, trauma, and tumors can result in the absence of the vagina, causing significant physical and psychological distress for affected patients [115,116]. Traditional vaginal reconstruction methods typically rely on non-vaginal tissues, such as skin grafts or intestinal segments, which have functional limitations and differ significantly from the natural vagina in both morphology and histology [117]. These approaches often lead to complications, such as contracture or necrosis, further impacting patient outcomes. The rapid advancement of 3D bioprinting technology offers a promising solution to these challenges by providing more effective and sophisticated approaches to vaginal reconstruction. Biomimetic 3D printing of vaginal tissue using AVM bioink has been proposed as an innovative method for reconstructing vaginal tissue (Fig. 9A) [64]. AVM was transformed into bioink by blending it with 15 % gelatin and 3 % sodium Alg. The viability of BMSCs within the printed scaffolds was assessed using a live/dead assay following 3D printing (Fig. 9B). In an in vivo assay, subcutaneous transplantation in rats was conducted using two groups: one with 3D printed scaffolds alone and the other with 3D printed scaffolds encapsulating CM-Dil-labeled BMSCs. Histological analysis through HE staining, immunohistochemistry, and immunofluorescence staining demonstrated that the 3D scaffolds containing BMSCs significantly enhanced vascularization and epithelialization of the printed vaginal tissue. Additionally, the BMSCs differentiated into vaginal epithelial-like and endothelial-like cells, indicating their potential role in promoting tissue regeneration and functionality.

### 3.6 Cervix tissue bioprinting

The human uterine cervix is a complex organ that undergoes significant structural changes during pregnancy and childbirth. It functions as a valve, keeping the fetus in the uterus until full term and aiding in delivery during labor. The cervix is primarily composed of fibrous connective tissue, with an ECM rich in collagen, elastin, and proteoglycans. Its cellular components include epithelium, fibroblasts, smooth muscle, and blood vessels [118,119]. The composition of the cervix varies, with the distal portion having a higher proportion of connective tissue, while the area near the myometrium contains more smooth muscle.

Cervical cancer is a prevalent malignancy in the female reproductive system and is one of the most frequently treated conditions by gynecologists. It can significantly impact a woman's ability to maintain pregnancy, with approximately 91 % of cases linked to human papillomavirus (HPV) infection [120,121]. It ranks second in mortality among female cancers, following breast cancer. Radical transabdominal hysterectomy is the most effective treatment, offering a survival rate of up to 90 % [121].However, procedure like cervical conization, which removes part of the cervix, can compromise cervical integrity, leading to increased risks of infertility, miscarriage, and preterm birth in treated patients [122,123]. Recently, 3D printing technology has been applied to develop personalized, implantable cervical device with drug-release functionality (Fig. 9C) [59]. These implants feature a cone-shaped design with hierarchical porous structures, developed based on clinical data, and are fabricated using PU through LDM, followed by lyophilization (Fig. 9D). Anti-HPV protein was incorporated into the porous structure under negative pressure techniques. The combination of elastic biomedical PU and the porous design mimicked the mechanical properties of natural cervical tissue. Cytotoxicity tests confirmed that the 3D printed implants supported cell adhesion and growth. The controlled pore structure also enabled precise loading and release of anti-HPV proteins, effectively inhibiting viral activity near the cervix. Additionally, bioprinting endometrial mesenchymal stem/stromal cells (eMSCs) onto a mesh offers a potential therapeutic approach for treating pelvic organ prolapse (POP) [82]. In this strategy, eMSCs encapsulated in a hydrogel were bioprinted onto a 3D melt electrospun (MES) mesh to create a tissue-engineered construct (Fig. 9D). An Aloe vera-sodium Alg composite hydrogel, optimized to a 1:1 ratio, was used for this purpose, and purified eMSCs from human endometrial biopsies were printed onto the MES meshes. In vivo assessments in immunodeficient mice showed that eMSC-printed MES constructs promoted tissue integration, enhanced eMSC retention, and induced an anti-inflammatory M2 macrophage response, as evidenced by F4/80+CD206+ colocalization. This approach presents a promising alternative for vaginal wall repair and POP treatment.

## 4 Challenges

### 4.1 Bioink and material challenges

The development of suitable bioinks remains a major bottleneck in 3D bioprinting of female reproductive tissues, as each organ exhibits distinct ECM compositions and mechanical properties, making it difficult to design universal bioinks that support tissue-specific cell functions and structural fidelity [124]. Natural hydrogels such as collagen, gelatin, and Alg are widely used due to their biocompatibility and ECM-mimicking properties, but they often lack sufficient mechanical strength and batch-to-batch consistency, which can compromise construct stability and reproducibility. Natural hydrogels such as collagen, gelatin, and alginate are widely used due to their biocompatibility and ECM-mimicking properties, but they often lack sufficient mechanical strength and batch-to-batch consistency, which can compromise construct stability and reproducibility. For example, collagen hydrogels (2–9 mg/mL) typically exhibit Young's modulus values of 0.5–16.6 kPa [125,126], gelatin hydrogels around 13–78 kPa, and alginate gels up to 70 kPa depending on crosslinking density [127]. In contrast, the mechanical modulus of native reproductive tissues such as the human endometrium is approximately 10–30 kPa [128], while the human ovary ranges from 3 to 10 kPa [129]. This mismatch highlights the difficulty of designing hydrogels that can simultaneously replicate the mechanical environment and support functional tissue regeneration. Synthetic polymers like PEG offer tunable mechanical properties and improved printability, yet may not provide the necessary biological cues for cell differentiation and function [130].

dECM bioinks, especially those derived from reproductive tissues, have emerged as promising candidates due to their ability to recapitulate organ-specific biochemical environments. These bioinks preserve key matrix proteins, growth factors, and glycosaminoglycans, promoting epithelial-stromal interactions, follicular development, and endometrial regeneration. However, several limitations hinder their widespread application. First, the decellularization process itself introduces variability depending on tissue source, species, age, and decellularization protocol, affecting the retention of bioactive components. Second, the immunogenic potential of incompletely decellularized matrices raises safety concerns for clinical translation. Third, dECM bioinks face reproducibility and scalability issues due to their inherent heterogeneity and limited availability of high-quality donor tissues, especially for human-derived reproductive dECMs [90,131].

### 4.2 Technical and structural limitations in bioprinting

Despite advances in bioprinting hardware and software, replicating the intricate architecture and function of female reproductive tissues remains challenging. Extrusion-based bioprinting, the most widely employed method in reproductive tissue engineering, is favored for its compatibility with a broad range of bioink viscosities and its capacity to fabricate large, cell-laden constructs. This approach offers resolutions ranging from 5 μm to several millimeters, depending on nozzle diameter and applied pressure. However, it typically operates at lower resolution (>100 μm in most practical settings) and imposes mechanical shear stress on encapsulated cells, which can compromise cell viability and phenotypic stability, particularly in delicate tissues such as the endometrium or ovarian follicles [132]. Reported cell viability for extrusion bioprinting generally ranges from 40 % to 80 %, with significant reductions observed as shear stress increases. For instance, using a 150 μm nozzle, raising the pressure from 5 psi to 40 psi led to a ∼38.75 % drop in cell viability, and exceeding shear stress of 150 kPa resulted in viability falling below 50 % [43,133]. Inkjet bioprinting offers improved resolution (∼50 μm) and induces minimal shear-related damage, making it suitable for patterning thin layers and vascular features. Thermal inkjet printing systems achieve resolutions around 50 μm (300 dpi) and have reported cell viabilities of approximately 75 % for primary embryonic neurons post-printing. Additionally, piezoelectric drop-on-demand systems allow modulation of droplet size and velocity through voltage adjustments, thereby controlling the mechanical stress imposed on cells. Even at high voltages (up to 80 V), cell viability remains above 90 % [134], supporting inkjet printing's potential for maintaining cellular integrity. Laser-assisted bioprinting, in contrast, achieves exceptional spatial resolution—up to ∼1 μm—enabling precise single-cell placement and fine control over 3D architecture. Notably, Laser-assisted bioprinting demonstrates superior cell viability across studies, typically exceeding 80 % and reaching as high as 95–100 % in optimized systems. This high viability is attributed to precise control of laser energy and the use of liquid intermediate layers (20–100 μm thick) that buffer cells from direct photothermal damage during printing [134,135]. However, laser-assisted bioprinting's high cost and technical complexity currently limit its broader implementation [43].

One of the most persistent challenges in reproductive tissue engineering is the bioprinting of constructs that are simultaneously multi-layered, vascularized, and hormonally responsive. For instance, while significant progress has been made in fabricating ovarian constructs [56], many of these models still fall short of supporting complete follicle maturation, primarily due to insufficient vascular integration and the inability to fully replicate the complex stromal and endocrine microenvironment of the native ovary. Similarly, attempts to bioprint the cervix or oviduct have been limited by difficulties in sourcing specialized epithelial cells and replicating their complex tubular or stratified structures. Long-term tissue maturation and function in vitro also remain major hurdles, as post-printing protocols are insufficient to fully recapitulate the dynamic physiological environment of the female reproductive tract.

### 4.3 Translational and clinical barriers

Translating bioprinted reproductive tissues from bench to bedside remains a significant challenge due to numerous clinical, regulatory, and logistical barriers. Standardization and reproducibility are major concerns, as variations in bioink composition, cell sourcing, and printing protocols can lead to inconsistent outcomes and complicate regulatory approval. Moreover, the lack of validated large-scale manufacturing processes and quality control standards continues to limit clinical scalability.

Ethical and regulatory issues are particularly pronounced in reproductive tissue engineering. The use of human-derived cells, dECM, or stem cells raises concerns regarding donor consent, immunogenicity, and long-term safety. While 3D bioprinting has advanced to preclinical and even early clinical stages in tissues such as cartilage and skin, there are currently no reports of successful clinical translation in female reproductive tissues, which remain among the most complex and sensitive targets for bioprinting [136]. Although constructs such as bioprinted endometrial or ovarian tissues have shown promise in animal models, none have yet progressed to clinical approval.

## 5 Future directions

### 5.1 Advanced future 3D bioprinting models in reproductive system

The application of 3D bioprinting is revolutionizing our understanding of complex physiological processes in the female reproductive system. By incorporating multiple cell types and tissue layers, 3D bioprinting has advanced the development of personalized in vitro models, which offer significant potential for disease modeling and therapeutic applications. Initially applied in fields such as orthopedics and plastic surgery, 3D printing has now entered reproductive medicine, providing new insights and innovative approaches for fertility preservation, gynecological conditions, and hormone replacement therapies.

Bioprinting technologies are evolving to incorporate body-on-a-chip platforms, which can model systemic disease like polycystic ovary syndrome (PCOS) and preeclampsia. To strengthen the translational relevance of bioprinting technologies, the development of a body-on-a-chip platform represents a promising direction for modeling systemic diseases that impact reproductive function. A functional multi-organ system could integrate bioprinted models of the ovary, uterus (including the endometrium and myometrium), and placenta, along with key metabolic and endocrine organs such as the liver, kidney, and hypothalamus–pituitary axis. These components are essential for recapitulating the hormonal feedback loops and metabolic interactions that regulate menstrual cycles, pregnancy, and reproductive aging [137]. For example, co-culturing an ovarian construct with a liver module would allow the evaluation of drug metabolism and its effects on folliculogenesis or steroidogenesis [138]. Similarly, integrating a uterine model with a placental unit and kidney module could facilitate the study of preeclampsia, which involves immune, vascular, and renal dysfunction [139]. Microfluidic connections mimicking physiological circulation would be critical for enabling inter-organ crosstalk, while AI-assisted control systems could dynamically adjust hormone concentrations and perfusion parameters to simulate cyclic hormonal changes. Such a platform could serve as a powerful tool for investigating reproductive toxicology, endocrine-disrupting chemicals, and complex disorders such as PCOS or preterm labor in a patient-specific manner.

Despite recent advancements, significant challenges persist in developing fully functional reproductive organs. Although there have been partial breakthroughs, such as biologically derived hormone replacement therapies for menopause and 3D-printed structures that replicate key reproductive tissues, the bioengineering of ovarian aging and fallopian tubes, both essential to reproductive health, remains largely underexplored. Ovarian aging, a natural process characterized by a decline in both the quantity and quality of oocytes, typically begins around age 35 and is a major contributor to age-related infertility [140,141]. Further research is needed to understand the biology, mechanisms, and genetic factors underlying ovarian aging, as well as to develop innovative interventions to mitigate its effects. Similarly, research into the bioengineering of fallopian tubes is limited, despite their essential role in gamete transport, fertilization, and early embryonic development [142,143]. Artificial fallopian tubes on a chip could provide a more physiologically relevant environment, improving fertility preservation strategies and enhancing in vitro fertilization (IVF) outcomes. In addition to ovarian aging and the fallopian tubes, other reproductive tissues such as the cervix and placenta have received limited attention in current bioprinting research. The cervix presents specific challenges due to its complex structural and biomechanical properties essential for maintaining pregnancy, while the placenta requires accurate reproduction of its highly vascularized and immunologically active microenvironment. Although preliminary studies have explored these tissues, the development of physiologically relevant and reproducible bioprinted models remains insufficient, highlighting an important gap in the field.

### 5.2 Vascularization and innervation in female reproductive bioprinted tissues

Vascularization is critical for modeling female reproductive organs due to the cyclic angiogenesis that occurs in the uterus and associated tissues. The endometrium undergoes continuous vascular remodeling, regulated by E2-driven microvascular expansion and P4-induced vessel stabilization post-ovulation [144]. Spiral arteries adapt dynamically to support implantation and pregnancy, while VEGF-mediated angiogenesis facilitates rapid endometrial repair following menstruation. Without vascular integration, in vitro models fail to replicate implantation and pregnancy conditions, limiting their physiological relevance. Angiogenesis is also essential in the ovary, where blood vessels regulate follicular development and corpus luteum function, and in the oviduct, where a dense capillary network sustains embryo transport [145].

Ahn et al. developed a vascularized UdECM hydrogels to enhance endometrial regeneration and fertility [66]. Their findings demonstrate that UdECM administration restores endometrial thickness, upregulates angiogenesis-related genes, and improves implantation success. Additionally, UdECM influences immune modulation by altering NK cell subpopulations, reducing cytotoxicity, and supporting pregnancy maintenance. These results highlight the necessity of incorporating vascularization strategies in reproductive tissue engineering to mimic the natural uterine microenvironment effectively. Endothelial cell co-cultures enhance angiogenesis in bioprinted ECs, improving nutrient delivery and implantation studies. Similarly, vascularized ovarian constructs support follicular survival and steroidogenesis, advancing fertility preservation strategies.

Beyond vasculature, innervation is critical in uterine and cervical models, where neurovascular interactions regulate function. Neurogenic bioinks enriched with growth factors facilitate nerve integration, improving functional tissue maturation [146,147]. Nerve innervation plays a critical role in regulating key functions of the female reproductive system, including uterine contractility, ovarian follicle development, cervical remodeling, and pain modulation. Both autonomic and sensory nerves interact with the endocrine and vascular systems to coordinate hormone secretion, immune responses, and tissue remodeling throughout the menstrual cycle. For instance, innervation of the uterus and ovaries influences essential processes such as implantation, ovulation [148]. Disruptions in neural signaling have been linked to reproductive disorders such as dysmenorrhea and preterm labor. Despite its importance, innervation is often overlooked in current bioprinting strategies. Incorporating neurogenic components, such as neural cells or growth factors, into bioprinted reproductive tissues could enhance their physiological relevance and functional integration.

### 5.3 AI integration for personalized female reproductive precision bioprinting

AI is emerging as a powerful tool in 3D bioprinting, with the potential to address persistent challenges in female reproductive tissue engineering, including vascularization, spatial organization, and scalability [149]. Traditional bioprinting approaches often struggle to achieve precise microarchitectures and consistent functionality. By integrating AI into bioprinting workflows, parameters such as bioink viscosity, extrusion pressure, and print speed can be dynamically optimized in real time, improving reproducibility and construct fidelity. Machine learning (ML) algorithms further contribute by analyzing large bioprinting datasets to predict scaffold integrity, optimize cell viability, and guide material selection [[150], [151], [152]].

**Fig. 10:**
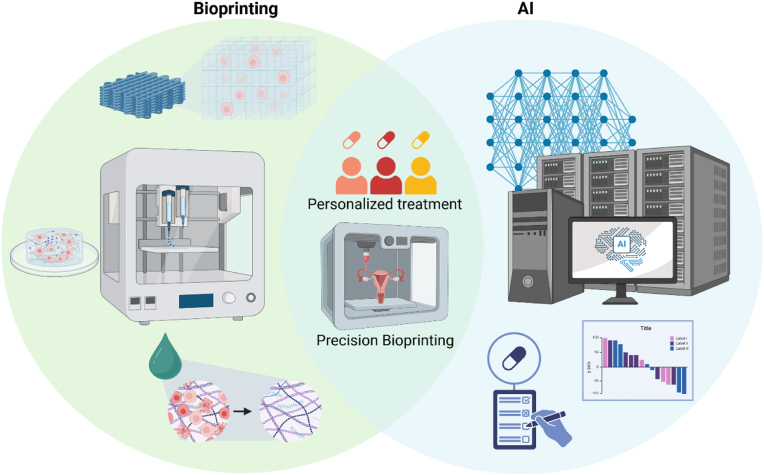
The schematic illustration of the integration of bioprinting and artificial intelligence (AI) for precision medicine. Bioprinting enables the fabrication of complex tissue structures using bioinks containing cells and biomaterials. AI-driven analysis enhances precision bioprinting by optimizing printing parameters, predicting outcomes, and facilitating personalized treatment strategies. The synergy between AI and bioprinting contributes to advancements in regenerative medicine and patient-specific therapies. Reproduced from under [153] an open-access license, ©2022 Bioprinting.

Recent developments have demonstrated the feasibility of closed-loop systems for enhanced print control. For example, Wenger et al. implemented a proportional-integral-derivative (PID) flow-regulated control system for pneumatic extrusion bioprinting. This approach automatically adjusts extrusion pressure based on real-time liquid flow feedback, enabling consistent output across a variety of bioinks [153] (Fig. 10).

Beyond parameter control, AI also facilitates predictive modeling of cell-material interactions, enabling virtual screening of biofabrication outcomes. Rafieyan et al. developed ML and deep learning platforms capable of classifying scaffold quality, predicting print fidelity, and evaluating cell behavior in silico [154]. Similarly, Joaquin et al. introduced a Python-based numerical model simulating Ca^2+^ ion diffusion and hydrogel crosslinking kinetics, advancing precision in vascular scaffold formation, an essential factor for ovarian and endometrial engineering [155]. These approaches reduce material waste, accelerate design iterations, and support scale-up for translational applications.

In high-throughput reproductive tissue bioprinting, AI-guided droplet systems have enabled precise volumetric control of cell-laden bioinks. Shin et al., developed an ML-powered bioprinting platform that adjusts nozzle size, viscosity, and pressure to generate uniform constructs. This technology is particularly promising for ovarian models, where accurate spatial organization of follicles and stromal cells is critical for functional outcomes [156].

Looking ahead, AI is expected to drive the development of autonomous and adaptive bioprinting platforms. Advances in multi-scale tissue modeling, real-time error correction, and generative design algorithms will support the fabrication of dynamic organoids capable of mimicking physiological processes, such as the cyclic remodeling of the endometrial lining. These innovations may transform in vitro implantation models and improve infertility treatments.

The convergence of AI, patient-specific bioinks, and multi-material bioprinting is poised to advance reproductive tissue engineering. AI-assisted vascular network design, combined with microfluidic bioprinting, may overcome current limitations in perfusion, particularly in endometrial and placental constructs. Generative AI can also facilitate the design of biomimetic tissues with appropriate mechanical strength, hormonal responsiveness, and cellular heterogeneity. In addition, the integration of patient-derived cells with AI-enhanced design could lead to personalized reproductive models for fertility restoration and individualized embryo-implantation predictions.

However, important challenges remain. Ensuring data privacy is essential, especially when working with patient-derived datasets. Model bias, resulting from imbalanced or non-representative training data, may limit prediction accuracy and clinical generalizability. Furthermore, the lack of standardized, high-quality datasets in reproductive bioprinting continues to restrict the development of robust and validated AI models. These limitations must be addressed through secure data governance, regulatory harmonization, and comprehensive clinical validation frameworks.

Altogether, the integration of AI into 3D bioprinting represents a promising direction for regenerative gynecology. It has the potential to improve precision, scalability, and personalization in reproductive tissue engineering and may ultimately redefine clinical approaches in fertility restoration and reproductive health.

### 5.4 Commercialization and translational outlook

Although 3D bioprinting of female reproductive tissues remains largely in the preclinical stage, recent developments signal growing interest in commercial and translational applications. Startups and academic spin-offs are exploring personalized bioinks, patient-derived cell therapies, and organ-on-a-chip platforms that simulate the reproductive microenvironment. For example, organoid-based endometrial models are being adapted for use in fertility screening and drug toxicity testing [157], while bioprinted vaginal scaffolds have progressed to early-stage animal studies with potential for reconstructive surgery. However, no fully bioprinted reproductive tissue has yet reached regulatory approval or clinical use, reflecting significant translational hurdles. These include the need for Good Manufacturing Practice (GMP)-compliant bioinks, long-term safety and integration studies, and standardized bioprinting protocols. Regulatory frameworks, such as the FDA Modernization Act 3.0 [158], have begun to accommodate in vitro tissue models as alternatives to animal testing, which may accelerate the path toward commercial adoption. Collaborations between academic institutions, industry partners, and regulatory agencies will be essential for bridging the gap between bench-scale innovation and bedside application. With ongoing progress in AI-assisted design, vascular integration techniques, and individualized biofabrication, bioprinted reproductive tissues are increasingly positioned to serve as effective platforms for drug screening, regenerative treatments, and tailored reproductive healthcare.

## 6 Conclusion

3D bioprinting has emerged as a transformative technology in female reproductive tissue engineering, offering unprecedented precision in replicating the structural, cellular, and functional complexities of reproductive organs. This review provides a comprehensive, organ-specific analysis of 3D bioprinting applications across the entire female reproductive system, including the ovary, endometrium, cervix, vagina, and placenta. Key current challenges are examined, including material-related issues, technical and structural limitations of bioprinting platforms, and barriers to clinical translation. In addition, this review explores advanced future directions in reproductive tissue bioprinting, with emphasis on vascularization and innervation strategies, the integration of AI for personalized precision bioprinting, and the commercialization landscape and translational outlook of this rapidly evolving field.

Building upon these insights, recent innovations in 3D bioprinting are enabling the development of physiologically relevant in vitro models that hold significant promises for restoring fertility, treating gynecological disorders, and advancing personalized reproductive medicine. Despite this rapid progress, several critical challenges persist, including the need for standardized organ-specific bioinks, and the translational gap between bench-scale constructs and clinical applications. Ultimately, the integration of bioprinting, regenerative medicine, and precision engineering offers a promising frontier for reproductive health, with bioprinted tissues poised to advance both our understanding of reproduction and the development of future therapies.

## CRediT authorship contribution statement

**Heesuh Yi:** Writing – original draft, Visualization. **Gaeun Lee:** Writing – original draft, Visualization. **Sanghyeok Park:** Visualization. **Juhyeong Ha:** Visualization. **Dayeong Choi:** Visualization. **Jihoon Ko:** Writing – review & editing, Writing – original draft. **Jungho Ahn:** Writing – review & editing, Writing – original draft.

## Declaration of competing interest

The authors declare no competing interests.

## Data Availability

No data was used for the research described in the article.
